# Associations of FTO and CLOCK Genetic Variants with Emotional Eating and Reward-Related Appetite Regulation Among Healthy Young Adult Males: An Exploratory Secondary Analysis

**DOI:** 10.3390/nu18030400

**Published:** 2026-01-26

**Authors:** Julie E. Brown, Christopher P. Hedges, Lindsay D. Plank, Andrea J. Braakhuis

**Affiliations:** 1 The Discipline of Nutrition, School of Medical Sciences, Faculty of Medical and Health Sciences, The University of Auckland, Auckland 1023, New Zealand; c.hedges@auckland.ac.nz (C.P.H.); a.braakhuis@auckland.ac.nz (A.J.B.); 2 Department of Surgery, Faculty of Medical and Health Sciences, The University of Auckland, Auckland 1023, New Zealand; l.plank@auckland.ac.nz

**Keywords:** FTO rs9939609, CLOCK rs1801260, emotional eating, food cravings, appetite regulation, genetic variation

## Abstract

**Background:** Patterns of dysregulated eating, including overeating, frequent snacking, and heightened food cravings, are associated with an increased risk of obesity and metabolic disease. Eating behaviors are multidimensional and can influence many factors, including social, cultural, and biological processes. Emerging evidence suggests that genetic variation may contribute to inter-individual differences in appetite regulation and reward-related eating, potentially influencing susceptibility to dysregulated eating patterns and behaviors. **Objectives:** This exploratory, secondary analysis investigated possible relationships between the genetic variants FTO rs9939609, CLOCK rs1801260, MC4R rs17782313, and CD36 rs1761667 and eating behavior traits and postprandial appetite regulation in healthy young males. **Methods:** Thirty healthy males (27.7 ± 3.6 y; BMI 24.5 ± 2.7 kg/m^2^) completed the Three-Factor Eating Questionnaire (TFEQ-R18) and consumed a standardized burrito-style meal, with appetite tracked over four hours using visual analogue scales (VAS). VAS data were baseline-adjusted and summarized as incremental area under the curve (AUC) to generate two derived exploratory composites of appetite suppression and cravings suppression. Genotyping was performed using iPLEX MassARRAY, and associations were tested with ANOVA and linear regression models. **Results**: FTO rs9939609 was significantly associated with higher emotional eating scores (β = 11.67; 95% CI 3.50, 19.83; *p* = 0.007, unadjusted), and this association remained significant after false discovery rate (FDR) correction. CLOCK rs1801260 showed a nominal association with reduced postprandial cravings suppression (β = −59.17; 95% CI −104.98, −13.35; *p* = 0.013, unadjusted). No associations were observed for MC4R or CD36. **Conclusions:** This exploratory analysis found a strong association between FTO rs9939609 and emotional eating, as well as a nominal relationship between CLOCK rs1801260 and craving regulation. These findings should be interpreted as hypothesis-generating and require confirmation in larger cohorts.

## 1. Introduction

In many developed countries, food is easy to access, yet eating patterns differ markedly, with some individuals struggling with strong cravings, frequent snacking, or overeating, while others maintain more stable and regulated patterns of intake [1]. These differences reflect the fact that eating behavior is multidimensional, shaped by a combination of influences [2]. Social and cultural norms, everyday food environments, and habitual practices all play a role, alongside biological factors that influence appetite and food preference [3,4,5]. Population-based studies have shown that this broader context is important when interpreting variations in appetite and food choice [6]. From a biological perspective, variation in eating behavior has also been linked to physiological processes and inherited genetic differences involved in appetite regulation and responses to food [3]. Central to this variability is the interaction between homeostatic mechanisms that regulate energy balance and hedonic systems that drive food reward [7].

The homeostatic system involves signaling processes along the gut–brain axis to modulate hunger and satiety and maintain energy balance, which is driven by hormones such as ghrelin, leptin, glucagon-like peptide-1 (GLP-1), cholecystokinin (CCK), and peptide YY (PYY) [8,9,10]. Alongside homeostatic regulation, the hedonic reward-driven system is mediated by mesolimbic dopamine signaling and responds to the rewarding properties of palatable foods, which can override physiological satiety cues [7]. Disruptions in either of these signaling systems can lead to impaired appetite regulation, promoting overconsumption of food, and result in dysregulated eating behaviors, contributing to obesity [11].

Research shows single nucleotide polymorphisms (SNPs) influence appetite regulation, taste perception, metabolic pathways, and susceptibility to obesity and related chronic diseases [12]. Such genetic predisposition may partly explain inter-individual differences in eating behavior and vulnerability to dysregulated intake patterns [10,11,12,13]. Our recent scoping review synthesized evidence from genome-wide association studies (GWAS) and candidate-gene studies of eating behavior traits and was undertaken to serve as the primary hypothesis-generating framework for the present work [13,14,15,16]. From this review, several SNPs consistently emerged as being associated with eating-related phenotypes. These included the Melanocortin 4 Receptor (MC4R) rs17782313, linked to central appetite regulation and satiety [9,17,18,19,20]; the Fat Mass and Obesity-Associated (FTO) rs9939609, associated with adiposity and eating behavior traits such as emotional eating (EE) and food cravings [21,22,23]; the Circadian Locomotor Output Cycles Kaput (CLOCK) rs1801260, reported in relation to circadian influences on appetite and meal timing [24,25,26]; and the Cluster of Differentiation 36 (CD36) rs1761667, associated with fat taste sensitivity and dietary fat preference [27,28,29,30].

Collectively, these variants have been implicated in neural reward pathways and appetite control, potentially modifying hedonic responses to food [3,9,31].

Despite this growing literature, much of the existing research has focused on mixed or female-dominant cohorts, leaving a gap in knowledge about young males—a group at risk of developing unhealthy eating patterns and metabolic complications during a key life stage [32,33,34]. Therefore, this secondary analysis conducted an exploratory investigation to examine whether selected genetic variants were individually associated with eating behavior traits and short-term postprandial appetite regulation in a small male cohort, using parallel single-SNP analyses.

### Aim

This exploratory secondary analysis examined whether four candidate SNPs, FTO rs9939609, MC4R rs17782313, CLOCK rs1801260, and CD36 rs1761667, previously identified as genetic modifiers of eating behavior, were associated with eating behavior traits, cognitive restraint (CR), emotional eating (EE), and uncontrolled eating (UE), as well as postprandial appetite responses in healthy young males.

## 2. Methods and Materials

### 2.1. Study Design and Setting

This secondary cross-sectional analysis used eating behavior data and within-day postprandial response measures from a parent double-blind, randomized trial conducted at the University of Auckland Clinical Research Centre (Auckland, New Zealand) between October and December 2020. The parent study investigated postprandial amino acid availability and appetite responses following a standardized meal [35]. An ancillary exploratory genetic study was also conducted to assess inter-individual variability in fasting and postprandial metabolic responses [36]. These genetic and eating behavior data were not primary outcomes of the parent study; therefore, we have not conducted a sample size calculation and accept the exploratory nature of our findings. The parent study included four test meals administered in a computer-generated random order, with participants and clinical staff blinded to allocation. Because outcome responses were similar across all meals, we selected the grain-fed beef mixed meal data for this secondary analysis.

Ethical approval for the parent study was granted by the New Zealand Ministry of Health’s Health and Disability Ethics Committees (Ref: 19/STH/226/AM02), and the study adhered to the principles outlined in the 1964 Declaration of Helsinki. All participants provided written informed consent, including consent for future secondary analyses of their data. The parent trial was registered at ClinicalTrials.gov (Identifier: NCT04545398; November 2020, https://clinicaltrials.gov/study/NCT04545398; accessed 15 March 2025). Participants were not involved in the design, conduct, or reporting process of the parent trial, ancillary genetic study, or this secondary analysis.

### 2.2. Participant Recruitment and Eligibility Criteria

As described previously [35], 30 healthy males aged 20–34 years were recruited from the community. Participants represented diverse ethnic backgrounds (see Supplementary Data). Eligibility criteria for this secondary analysis were identical to those for the parent study. Participants were required to report good general health, follow an omnivorous diet, and have a body mass index (BMI) ≤ 30 kg/m^2^.

Exclusion criteria included chronic disease, hyperlipidemia, regular prescription medication use (other than occasional NSAIDs/antihistamines), a history of anosmia or ageusia, current dieting or disordered eating, smoking, or recreational drug use [37]. Baseline procedures and anthropometric measurements were performed as previously reported [35].

### 2.3. Eating Behavior Assessment (TFEQ-R18)

The dietetic team administered the TFEQ-R18 at screening, a validated 18-item questionnaire adapted from the original 51-item TFEQ, to assess CR, UE, and EE [37,38]. Items were rated on a 4-point Likert scale (1 = definitely true, 2 = mostly true, 3 = mostly false, 4 = definitely false). CR was assessed by items 2, 11, 12, 15, 16, and 18 (e.g., “I deliberately take small helpings as a means of controlling my weight”); UE by items 1, 4, 5, 7, 8, 9, 13, 14, and 17 (e.g., “Sometimes when I start eating, I just can’t seem to stop”); and EE by items 3, 6, and 10 (e.g., “When I feel anxious, I find myself eating”).

Raw domain scores for each domain were rescaled to a 0–100 percentage scale using the formula: [(raw-score- lowest possible raw score)/raw score range] × 100. Higher scores indicated greater restraint, increased UE, or a stronger tendency towards EE.

### 2.4. Appetite Assessment and Standardized Test Meal

Subjective appetite was assessed following consumption of a standardized burrito-style meal (~817 kcal; 61 g protein; 30 g fat; 71 g carbohydrates; 9 g fiber) as previously described [36]. Appetite sensations were measured using a validated 100-point online VAS administered via Qualtrics XM software (version 2020, Provo, UT, USA) [39]. Participants rated hunger, satiety, fullness, PFC, and cravings for sweet, salty, savory, and fatty foods at baseline/pre-meal (t-pre; −1 h), immediately post-meal (t0), and at 30, 60, 120, 180, and 240 min postprandially (t30 to t240). Ratings were completed without knowledge of genotype.

Appetite-related questions included: “How hungry do you feel?” (0 = not hungry at all, 100 = very hungry); “How satisfied do you feel?” (0 = completely empty, 100 = very full); “How full do you feel?” (0 = not full at all, 100 = very full); and “How much do you think you can eat?” (0 = a lot, 100 = none at all). Craving-related questions were phrased as: “Would you like to eat something [sweet/salty/savory/fatty]?” (0 = yes, very much; 100 = no, not at all).

### 2.5. Composite Appetite and Cravings Suppression Scores

Two derived exploratory composite measures of appetite suppression and cravings suppression were calculated from repeated VAS ratings over the 0–240-min postprandial period to capture short-term appetite dynamics following the standardized test meal. Appetite suppression at each time point was calculated as the mean of four appetite-related VAS ratings: (satiety + fullness + PFC + (100 − hunger))/4, with hunger reversed so that higher scores reflected lower appetite and greater satiety [40]. Cravings suppression was calculated at each time point as the mean of the VAS ratings for sweet, salty, savory, and fatty food cravings, with higher scores indicating reduced cravings or greater suppression of food cravings. These derived composite measures are consistent with validated approaches used in previous research on appetite [40,41,42,43].

To capture postprandial dynamics over time, each composite time series was baseline-adjusted by subtracting the participant’s pre-meal (t-pre) value from all subsequent time points and then reduced to an incremental area under the curve (AUC; t0 to t240) using the trapezoidal method [44]. This yielded two derived exploratory outcomes per participant: appetite suppression AUC and cravings suppression AUC, capturing cumulative postprandial change relative to fasting. All derived values are provided in the Supplementary Data.

### 2.6. Genetic Analysis and SNP Selection

Genotyping procedures have been previously reported [36]. Briefly, buccal DNA was collected using Isohelix SK-1 swab kits, supplied as part of a sample collection pack from Nutrigenomix Ltd. (Sydney, NSW, Australia). Samples were processed at Nutrigenomix Ltd. Laboratories (University of Sydney, NSW, Australia), a CLIA-certified and CAP-accredited facility (College of American Pathologists). Genotyping was conducted using the iPLEX Gold assay on the Sequenom MassARRAY platform (Agena Bioscience Inc., San Diego, CA, USA). Quality-control metrics met all platform recommendations for all SNPs (success rates > 97%, call rate > 98%, duplicate concordance > 99%, and missingness < 3%), confirming high reliability of the genetic data [45].

From the full Nutrigenomix panel, four SNPs were selected a priori for analysis (FTO rs9939609 T>A, CD36 rs1761667 G>A, MC4R rs17782313 T>C, and CLOCK rs1801260; 3111T>C), guided by our previous scoping review [14].

All four SNPs conformed to Hardy–Weinberg equilibrium (HWE) using chi-square analysis; however, given the small sample size (N = 30), HWE results should be interpreted cautiously due to limited power and small expected genotype counts (*p* > 0.05; see Supplementary Data). All SNPs were annotated using dbSNP build 153 [46], and aligned to the GRCh38 human genome reference build [47].

### 2.7. Data Analysis and Statistics

Analyses were performed using complete cases (N = 30), with no missing data. Descriptive statistics were reported as mean ± SD by outcome and genotype group. Baseline-adjusted incremental AUC values were calculated in Microsoft Excel using the trapezoidal method, and statistical analyses were conducted using IBM SPSS Statistics v30 with a two-tailed significance threshold of α = 0.05.

Genotype–phenotype associations were first examined using one-way ANOVA to compare mean scores across genotype groups (major-allele homozygote, heterozygote, minor-allele homozygote) for each SNP. The five outcomes included CR, UE, and EE from the TFEQ-R18 (rescaled 0–100), along with appetite suppression and cravings suppression derived from baseline-adjusted incremental AUC values. F statistics, degrees of freedom, two-sided *p* values, and effect sizes (η^2^ = SS between/SS total) were calculated and reported.

Additive linear regression models (coded 0, 1, 2 copies of the minor allele) were used to test dose–response trends. Both unadjusted and adjusted models (including age and BMI) were conducted, with results reported as unstandardized β coefficients, 95% confidence intervals (CI), *p* values, and R^2^ [48].

Analyses and reporting followed STREGA (STrengthening the REporting of Genetic Association studies) guidelines for genetic research [49]. Findings are presented with appropriate caution, reflecting the exploratory nature of the study, the limited sample size, and the absence of pre-specified genetic hypotheses. Results are therefore intended to support transparent reporting and interpretation rather than confirmatory inference.

Model assumptions were checked by Levene’s test for homogeneity of variance (*p* > 0.05), visual inspection of histograms, Q–Q, and P–P plots for residual normality, and residual-fitted plots for heteroscedasticity.

The Shapiro–Wilk test was used to assess normality, as it is appropriate for small sample sizes (<50) [50]. Multicollinearity was assessed using variance inflation factors (VIFs), with all VIFs < 2. For descriptive interpretation only, unstandardized β coefficients were additionally expressed as percent change relative to the sample mean (%Δ per allele = β/outcome mean × 100) following established guidelines [51].

### 2.8. Statistical Adjustment for Multiple Comparisons 

Multiple testing used the Benjamini–Hochberg false discovery rate (BH-FDR) procedure, applied in two ways: globally across all 20 tests (four SNPs × five outcomes), and separately for each SNP across the five outcomes (q = 0.05) [52].

Only associations that remained significant after BH-FDR correction were interpreted as statistically significant, while nominal (*p* < 0.05) findings were treated as exploratory.

## 3. Results

All 30 healthy young male participants were included in the final analysis (see Supplementary Data). Baseline characteristics and fasting appetite ratings are presented in Table 1. Large standard deviations for baseline VAS appetite measures are expected, as fasting subjective appetite ratings typically show wide inter-individual variability in healthy populations [39,53].

### 3.1. Allele Frequencies and Reliability

Allele frequencies and genotype distributions for the four SNPs are presented in Table 2, alongside reference population frequencies. All SNPs conformed to HWE (*p* > 0.05; see Supplementary Data). Reference frequencies were retrieved from gnomAD v4.1.0 and NCBI dbSNP databases [54,55].

The TFEQ-R18 showed good internal consistency for the total scale (Cronbach α = 0.79). Subscale reliabilities were acceptable for each domain: CR (α = 0.87), UE (α = 0.77), and EE (α = 0.68) [57,58,59]. Internal consistency for appetite-related VAS items was acceptable (Cronbach α = 0.75), with subscale reliabilities α values ranging from 0.68 to 0.73 [57].

### 3.2. Descriptive Analysis

Descriptive statistics for eating behavior traits and postprandial appetite-related outcomes are provided in Table 3. CR, UE, and EE were derived from the TFEQ-R18 scores and rescaled to percentages. Appetite suppression and cravings suppression composites were derived as baseline-adjusted incremental AUCs from t0 to t240 measures, as described in the Methods.

### 3.3. Genotype Group Comparisons (One-Way ANOVA)

One-way ANOVA identified nominal genotype differences for FTO rs9939609 on EE, and for CLOCK rs1801260 on UE and cravings suppression (Table 4). MC4R rs17782313 showed non-significant trends for CR and cravings suppression, while CD36 rs1761667 showed no evidence of genotype differences for any outcome.

These ANOVA results are reported descriptively and interpreted as exploratory, with the findings further evaluated in subsequent linear regression analyses that incorporate correction for multiple testing. Levene’s tests were non-significant for all outcomes (*p* > 0.05), indicating homogeneity of variance across genotype groups and supporting the use of one-way ANOVA.

### 3.4. Additive Linear Regression Models

Additive linear regression models were fitted for each SNP with eating behavior traits (CR, UE, EE) and postprandial appetite outcomes (appetite suppression and cravings suppression). Genotypes were coded as 0, 1, or 2 copies of the minor allele. Table 5 presents the unstandardized regression coefficients (β), 95% confidence intervals, and *p*-values for both unadjusted models and models adjusted for age and BMI.

Model diagnostics met assumptions: residual normality was assessed using visual inspection of histograms, Q–Q and P–P plots, and the Shapiro–Wilk test, with all tests non-significant (*p* > 0.05). Residuals-versus-fitted plots did not indicate heteroscedasticity, and VIFs < 2 indicated no problematic multicollinearity.

A nominal association was observed between CLOCK rs1801260 and cravings suppression, with each *C*-allele associated with lower postprandial suppression in both unadjusted and age- and BMI-adjusted models (Table 5). However, this association did not survive BH-FDR correction and is therefore reported for completeness and interpreted as exploratory. No consistent evidence of association was observed for MC4R rs17782313 or CD36 rs1761667 across behavioral or postprandial appetite outcomes. Across all models, the only association that remained statistically significant after correction for multiple testing was the relationship between FTO rs9939609 and EE. All other associations are reported descriptively and interpreted as nominal.

### 3.5. Multiple Testing Correction

When BH-FDR correction was applied across all 20 unadjusted tests (4 SNPs × 5 outcomes) no associations remained significant at the global level (global BH-FDR q > 0.10). When applying a per-SNP BH-FDR correction (q = 0.05) across the five outcomes for each SNP, only the association between FTO rs9939609 and EE remained significant (unadjusted q = 0.034; adjusted q = 0.045; see Supplementary Data). Accordingly, this association was considered significant, while all other findings were classified as exploratory.

### 3.6. Genotype–Phenotype Association: FTO rs9939609 and EE

**Figure 1:**
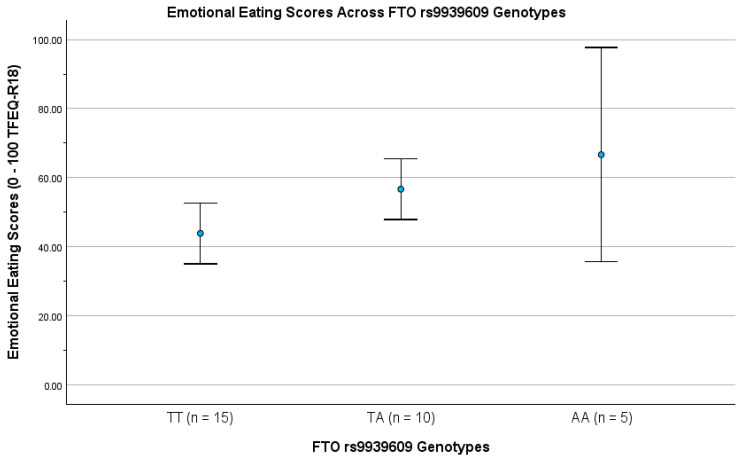
Emotional eating scores (0–100; ±95% CI) by FTO rs9939609 genotypes (TT, TA, AA). Higher scores reflect a greater tendency towards emotional eating.

The positive association between FTO rs9939609 and EE is shown in Figure 1. Each additional *A*-allele was associated with higher EE scores across genotypes (TT, TA, AA). This relationship was statistically significant in both ANOVA and regression analyses, remained significant after adjustment for age and BMI, and survived per-SNP BH-FDR correction.

In the unadjusted model, FTO explained 23.4% of the variance in EE within this cohort (R^2^ = 0.234).

### 3.7. Effect Size Interpretation

To aid interpretation of effect magnitude, unadjusted percent-change effect sizes (%Δ per allele relative to the outcome mean) were +22.47% for EE for FTO rs9939609. Additional results are provided in the Supplementary Data. This is provided for descriptive purposes only and should be interpreted with caution, given the exploratory nature of the analysis.

## 4. Discussion

This exploratory secondary analysis was informed by our prior scoping review of genetic influences on eating behavior [14] and examined whether common variants in FTO, MC4R, CLOCK, and CD36 were associated with eating behavior traits and postprandial appetite dynamics in healthy young males. The selection of these genetic variants was guided by evidence linking them to appetite regulation, satiety signaling, reward processing, and circadian control of food intake [13,26,29,60]. Therefore, this study employed a targeted candidate-gene approach to prioritize these variants based on their strong biological plausibility in relation to eating behaviors [1].

The key finding of this study was a statistically significant association between FTO rs9939609 and EE, with *A*-allele carriers displaying higher EE scores than TT homozygous genotype (*p* = 0.007, unadjusted). This association, corresponding to an approximate 22% increase in EE score per risk allele, remained significant after adjustment for age and BMI and following BH–FDR correction. This suggests that, within this cohort, the FTO *A*-allele may increase susceptibility to emotion-driven eating, supporting the behavioral relevance of this variant despite the modest sample size. This result is consistent with studies linking FTO to dysregulated eating, obesity-related phenotypes, and diet quality [26,61,62].

Previous studies have linked FTO to both homeostatic and reward-related regulation of food intake, with evidence suggesting involvement of hypothalamic pathways and mesolimbic dopaminergic circuits that govern food motivation and reward [63]. Variation within this gene may therefore increase sensitivity to palatable food cues and promote eating in response to emotional or situational triggers rather than physiological hunger alone. While neural and hormonal mediators were not directly measured in the present study, the association observed with EE is consistent with this broader biological framework and supports the view of FTO as a marker of behavioral vulnerability, rather than a gene that directly drives energy intake.

We also observed a nominal association between the CLOCK rs1801260 *C*-allele and lower postprandial cravings suppression, which did not remain significant after BH-FDR correction. This nominal finding should therefore be interpreted with caution and considered as an exploratory finding. Nevertheless, the direction of effect is consistent with prior evidence implicating CLOCK variants in disrupted circadian regulation of eating behavior and appetite timing [25,64,65]. Circadian misalignment has been associated with altered secretion of appetite-regulating hormones, including ghrelin and glucagon-like peptide-1 (GLP-1), which may promote increased hunger, snacking, and reduced satiety [6,43]. These observations suggest that variation in the circadian CLOCK gene may influence the temporal patterns of appetite regulation, particularly under conditions of disturbed sleep patterns or irregular eating schedules [66]. Although preliminary, our finding highlights the potential relevance of circadian biology in postprandial appetite responses and warrants further investigation in larger, adequately powered samples.

There were no significant associations detected for MC4R rs17782313 or CD36 rs1761667, consistent with previous reports suggesting that the effects of these variants on eating behaviors and food preferences may be difficult to detect in our small cohort [67]. It is also possible that the influence of these variants emerges more clearly in interaction with environmental exposures, dietary patterns, or broader polygenic risk, which were beyond the scope of the present analysis.

Taken together, these results suggest that variation in genes related to appetite and reward may contribute to inter-individual differences in eating behavior among young male adults [9,12]. Although this study focused on genetic factors, the observed associations should be interpreted within the wider behavioral context of eating behavior, which reflects an interplay between genetic predisposition, alongside psychological, social, and environmental influences [4].

### 4.1. Strengths and Limitations

This study combined validated behavioral measures, detailed postprandial appetite profiles, and targeted genotyping, providing an integrated assessment of the genotype-behavior relationship. However, we acknowledge several limitations. The sample size was small (N = 30), which limits statistical power, increases the risk of both Type I and Type II errors, and restricts the ability to adjust for potential confounders, including ethnicity [68]. Participants were limited to healthy young males, addressing a gap in the literature, and a strength of the study [69]. The cross-sectional nature of the analysis precludes causal inference regarding the direction of SNP–behavior relationships [70,71]. The self-reported questionnaires may introduce subjectivity and recall or response bias [40,72]. Lastly, although BH-FDR correction was applied, a residual risk of inflated Type I error (i.e., a *p*-value of >0.05) remains in small-sample studies with multiple tests [73,74]. These limitations are common to many exploratory nutrigenetic studies in small cohorts [75,76].

Despite these limitations, exploratory genetic studies such as this are valuable for generating biologically grounded hypotheses and informing the design of larger, well-powered trials [77]. In this context, this work contributes to this process by highlighting FTO as a variant of interest in relation to EE.

### 4.2. Future Directions

Future research could confirm the associations between genetic variation in SNPs related to eating behaviors, eating patterns, and subsequent health effects [78,79]. Eating behavior does not arise solely from genetics. Incorporating factors such as food environments, psychosocial stress, sleep patterns, and cultural eating practices alongside genetic data may further improve the interpretation of observed associations [4]. Interestingly, future studies may find it useful to examine whether tertile-based TFEQ-R18 groupings, together with genetic risk alleles, show similar patterns in other populations [80].

## 5. Conclusions

This exploratory hypothesis-generating study provides preliminary evidence that the FTO rs9939609 variant is associated with higher EE scores within this cohort. This association remained significant after correction for multiple testing. In contrast, the association observed between CLOCK rs1801260 and postprandial cravings suppression was nominal and did not survive BH-FDR correction and should therefore be interpreted with caution. Overall, these findings should be interpreted as exploratory and require confirmation in larger cohorts [81].

## Figures and Tables

**Table 1:** Participant characteristics and baseline visual analogue scale (VAS) appetite scores.

Characteristics (N = 30)	Mean ± SD
Age (years)	27.7 ± 3.62
Body weight (kg)	76.6 ± 10.0
Height (cm)	176.6 ± 5.80
Body mass index (BMI) (kg/m^2^)	24.5 ± 2.69
Hunger (VAS, 0–100)	72.7 ± 25.3
Satiety (VAS, 0–100)	32.4 ± 26.6
Fullness (VAS, 0–100)	34.4 ± 33.6
Prospective food consumption (VAS, 0–100)	29.2 ± 23.3
Desire for sweet foods (VAS, 0–100)	48.0 ± 28.1
Desire for salty foods (VAS, 0–100)	43.9 ± 19.1
Desire for savory foods (VAS, 0–100)	33.0 ± 22.8
Desire for fatty foods (VAS, 0–100)	52.1 ± 23.3

Data are presented as mean ± SD, reflecting the normal distribution of variables.

**Table 2:** Allele frequencies and genotype distributions for the four SNPs (N = 30).

**Gene**	**SNP rsID**	**Population**	**Allele Frequency**	**Genotype Frequency**		
FTO	rs9939609	Reference	T = 0.60, A = 0.40	TT = 42%, TA = 44%, AA = 13%		
FTO	rs9939609	Study cohort (N = 30)	T = 0.67, A = 0.33	TT = 15 (50%), TA = 10 (33%), AA = 5 (17%)		
CD36	rs1761667	Reference	G = 0.52, A = 0.48	GG = 27%, GA = 50%, AA = 23%		
CD36	rs1761667	Study cohort (N = 30)	G = 0.62, A = 0.38	GG = 11 (37%), GA = 15 (50%), AA = 4 (13%)		
MC4R	rs17782313	Reference	T = 0.77, C = 0.23	TT = 61%, CT = 34%, CC = 5%		
MC4R	rs17782313	Study cohort (N = 30)	T = 0.72, C = 0.28	TT = 15 (50%), CT = 13 (43%), CC = 2 (7%)		
CLOCK	rs1801260	Reference	T = 0.64, C = 0.36	TT = 54%, CT = 38%, CC = 7%		
CLOCK	rs1801260	Study cohort (N = 30)	T = 0.78, C = 0.22	TT = 19 (63%), CT = 9 (30%), CC = 2 (7%)		

Reference frequencies were retrieved from gnomAD v4.1.0 (https://gnomad.broadinstitute.org; accessed 15 March 2025) [54], and NCBI dbSNP (https://www.ncbi.nlm.nih.gov/snp; accessed 15 March 2025) [55,56], across multiple population groups. Percentages may not round to 100 due to rounding.

**Table 3:** Eating behavior and appetite-related measures.

Metric	Mean	SD	SE	95% CI (Lower)	95% CI (Upper)
Cognitive restraint (%)	50.42	16.30	3.03	44.23	56.61
Uncontrolled eating (%)	53.52	13.28	2.47	48.47	58.56
Emotional eating (%)	51.94	17.96	3.34	45.12	58.77
Appetite suppression (AUC)	154.09	106.99	19.53	115.80	192.38
Cravings suppression (AUC)	101.20	82.84	15.12	71.55	130.84

Eating behavior traits (CR, UE, EE) were derived from TFEQ-R18 questionnaire scores, rescaled to percentages. Appetite suppression was computed as the mean of satiety, fullness, PFC, and (100–hunger). Cravings suppression was computed as the mean of sweet, salty, savory, and fatty cravings. For each variable, VAS data were summarized as baseline-adjusted incremental AUC (t0–t240, trapezoidal method).

**Table 4:** One-way ANOVA results for genotype differences across eating-behavior outcomes (N = 30).

SNP (rs ID)	Outcome	F-Value	*p* Value	η^2^
FTO rs9939609	Emotional eating	4.16	0.027 *	0.236
CLOCK rs1801260	Uncontrolled eating	5.61	0.009 **	0.294
CLOCK rs1801260	Cravings suppression	3.63	0.040 *	0.212
MC4R rs17782313	Cognitive restraint	2.73	0.083	0.168
MC4R rs17782313	Cravings suppression	2.65	0.089	0.164
CD36 rs1761667	No significant differences observed			

Values are from one-way ANOVA with df = (2,27). η^2^ values calculated as SS between/SS total representing the proportion of variance explained by genotype. * *p* ≤ 0.05; ** *p* ≤ 0.01.

**Table 5:** Linear regression analysis for FTO rs9939609 T>A, CD36 rs1761667 G>A, MC4R rs17782313 T>C, and the CLOCK rs1801260 (3111T>C) SNPs with eating behavior outcomes (N = 30).

Outcome	SNP	β Unstd. (Unadjusted)	95% CI (Unadjusted)	*p* (Unadjusted)	β Unstd. (Adjusted)	95% CI (Adjusted)	*p* (Adjusted)
**Cognitive restraint**	FTO	2.13	[−6.30, 10.55]	0.610	2.04	[−6.39, 10.48]	0.623
	CD36	−6.02	[−15.18, 3.15]	0.189	−7.61	[−18.04, 2.82]	0.146
	MC4R	5.06	[−5.01, 15.12]	0.312	5.53	[−4.83, 15.89]	0.282
	CLOCK	−3.78	[−13.92, 6.37 ]	0.452	−2.96	[−13.46, 7.54]	0.567
**Uncontrolled eating**	FTO	3.94	[−2.78, 10.67]	0.240	3.76	[−2.79, 10.31]	0.249
	CD36	0.18	[−7.52, 7.88]	0.962	−1.49	[−10.10, 7.12]	0.726
	MC4R	1.58	[−6.75, 9.91]	0.701	2.72	[−5.62, 11.06]	0.508
	CLOCK	−3.54	[−11.78, 4.70]	0.387	−3.51	[−11.78, 4.75]	0.390
**Emotional eating**	FTO	11.67	[3.50, 19.83]	0.007 *	11.41	[3.58, 19.23]	0.006 *
	CD36	5.38	[−4.82, 15.59]	0.289	4.30	[−7.22, 15.81]	0.450
	MC4R	1.49	[−9.79, 12.77]	0.789	3.20	[−8.08, 14.47]	0.565
	CLOCK	−4.42	[−15.59, 6.74]	0.424	−4.58	[−15.74, 6.59]	0.407
**Appetite suppression**	FTO	−28.52	[−82.03, 24.99]	0.284	−27.27	[−81.12, 26.59]	0.308
	CD36	−21.28	[−81.73, 39.17]	0.477	−13.71	[−84.00, 56.58]	0.692
	MC4R	1.83	[−64.32, 67.98]	0.955	−5.88	[−74.55, 62.80]	0.862
	CLOCK	−4.45	[−70.58, 61.69]	0.891	−4.08	[−72.58, 64.42]	0.904
**Cravings suppression**	FTO	−10.60	[−52.70, 31.50]	0.610	−9.57	[−51.58, 32.44]	0.644
	CD36	−35.91	[−81.05, 9.24]	0.114	−25.18	[−78.33, 27.98]	0.339
	MC4R	30.27	[−19.59, 80.14]	0.224	22.84	[−29.09, 74.78]	0.374
	CLOCK	−59.17	[−104.98, −13.35]	0.013 *	−53.24	[−101.26, −5.23]	0.031

Appetite suppression was calculated from hunger, satiety, fullness, and prospective food consumption scores, and cravings suppression from sweet, salty, savory, and fatty scores. The primary models were unadjusted, with sensitivity analysis adjusted for age and BMI. Unstandardized coefficient (β), 95% CI, and *p* are reported. Asterisks (*) indicate statistically significant (*p* ≤ 0.05).

## Data Availability

The original contributions presented in this study are included in the article/Supplementary Material. Further inquiries can be directed to the corresponding author.

## Supplementary Materials

The following supporting information can be downloaded at: https://www.mdpi.com/article/10.3390/nu18030400/s1, Table S1. Ethnic distribution of participants (N = 30). Table S2. Area under the curve (AUC) calculations for fat craving scores (N = 30). Table S3. Three-factor eating questionnaire-R18 (TFEQ-R18) and appetite-related measures (N = 30). Table S4. Genotype distribution of four SNPs related to eating behaviors (N = 30). Table S5. Hardy-Weinberg Equilibrium (HWE) and chi-square calculations for four SNPs (N = 30). Table S6. Example Benjamini–Hochberg (BH) FDR calculation for FTO (5 outcomes, α = 0.05). Table S7. Example Benjamini–Hochberg (BH) FDR calculation for CLOCK (5 outcomes, α = 0.05). Table S8. Effect size interpretation (%Δ per allele relative to the outcome mean).

## Abbreviations

The following abbreviations are used in this manuscript

AUC	Area under the curve
BH-FDR	Benjamini–Hochberg false discovery rate
BMI	Body Mass Index
CCK	Cholecystokinin
CD36	Cluster of Differentiation 36
CI	Confidence interval
CLOCK	Circadian Locomotor Output Cycles Kaput
CR	Cognitive restraint
EE	Emotional eating
FTO	Fat Mass and Obesity-Associated
GLP-1	Glucagon-like peptide-1
GWAS	Genome-wide association studies
HWE	Hardy–Weinberg Equilibrium
MC4R	Melanocortin 4 receptor
PYY	Peptide YY
PFC	Prospective food consumption
SNP	Single nucleotide polymorphism
TFEQ	Three-Factor Eating Questionnaire
UE	Uncontrolled eating
VAS	Visual Analogue Scale
