# Supplementary material for: Associations of FTO and CLOCK Genetic Variants with Emotional Eating and Reward-Related Appetite Regulation Among Healthy Young Adult Males: An Exploratory Secondary Analysis
Source: Nutrients. 2026 Jan 26;18(3):400. doi: 10.3390/nu18030400 (PMC12899139; doi:10.3390/nu18030400)
Supplement: Supplementary file 1 [file nutrients-18-00400-s001.zip › nutrients-4049377-supplementary.pdf]

Tables S1 – S8

Table S1. Ethnic distribution of participants (N = 30)

**Ethnicities**	**Total**
Chinese	3
European	1
Filipino	1
Greek Australian	1
Indian	4
Iranian	1
Korean	3
Latin American	2
New Zealand European	8
NZ European, Māori, Chinese	1
Others (American)	1
Singaporean Chinese	1
Sri Lankan	1
UK	1
Vietnam	1
Total	30

Self-reported ethnicities of the 30 participants enrolled in the study.

Table S2. Area under the curve (AUC) calculations for fat craving scores (N = 30)

**Fat craving**	**#1**	**#2**	**#3**	**#4**	**#5**	**#6**	**#7**	**#8**	**#9**	**#10**	**#11**	**#12**	**#13**	**#14**	**#15**
Time															
-1	51	70	100	79	49	13	27	50	50	21	100	51	40	21	40
0	95	100	100	100	87	67	37	100	21	100	100	77	74	82	90
0.5	99	94	100	100	96	84	11	95	36	99	100	76	87	77	100
1	100	100	100	97	90	93	24	87	50	100	100	77	91	72	100
2	88	81	100	100	84	80	46	75	20	100	100	63	97	71	100
3	97	100	100	98	87	67	50	50	25	100	66	64	100	74	100
4	97	99	100	80	39	45	71	31	29	100	38	45	100	49	100
**AUC**	177.75	97.50	0.00	69.75	131.75	246.00	56.25	78.25	-79.75	315.50	-65.00	60.50	217.25	198.50	237.50

**Fat craving**	**#16**	**#17**	**#18**	**#19**	**#20**	**#21**	**#22**	**#23**	**#24**	**#25**	**#26**	**#27**	**#28**	**#29**	**#30**
Time															
-1	31	82	88	35	54	92	40	46	72	50	94	41	50	39	42
0	83	89	91	100	100	100	100	100	100	100	97	100	100	48	33
0.5	87	95	91	100	100	100	50	100	100	97	100	100	50	50	41
1	95	94	95	100	100	100	50	97	100	94	91	100	50	49	47
2	80	94	100	96	76	100	50	85	100	100	95	100	50	48	46

3	44	92	100	87	52	100	50	77	100	100	67	100	50	49	48
4	29	85	100	50	33	98	60	59	100	99	43	100	50	50	22
**AUC**	150.00	40.75	37.50	218.00	78.50	31.00	57.50	155.25	112.00	193.50	53.75	236.00	12.50	39.75	1.00

All reported AUC values were calculated from participants' visual analog scale (VAS) rating using the trapezoidal method in Excel, across time points at 0, 0.5, 1, 2, 3, and 4 h. Values were baseline-adjusted by subtracting the −1 h (t-pre) rating at each postprandial time point, and incremental AUCs were computed (areas above baseline positive, areas below baseline negative) to represent postprandial changes from fasting.

Table S3. Three-factor eating questionnaire-R18 (TFEQ-R18) and appetite-related measures (N = 30)

ID	Cognitive Restraint %	Uncontrolled Eating %	Emotional Eating %	Appetite suppression AUC	Cravings Suppression AUC
1	33.33	72.22	66.67	252.31	143.06
2	58.33	50.00	58.33	194.13	103.75
3	79.17	33.33	33.33	261.63	63.75
4	25.00	25.00	25.00	329.63	48.25
5	50.00	50.00	50.00	181.69	132.13
6	50.00	75.00	75.00	221.94	150.69
7	41.67	47.22	33.33	-20.06	80.38
8	29.17	58.33	66.67	204.38	88.25
9	54.17	55.56	50.00	-11.88	-132.50
10	70.83	38.89	25.00	217.19	285.88
11	33.33	52.78	33.33	301.19	79.19
12	66.67	69.44	50.00	167.00	99.69
13	37.50	47.22	25.00	211.13	215.69
14	62.50	55.56	50.00	82.38	135.13
15	66.67	55.56	91.67	208.81	226.19
16	37.50	75.00	50.00	103.31	146.69
17	58.33	69.44	75.00	155.81	145.25
18	83.33	44.44	75.00	-33.38	29.94
19	54.17	77.78	66.67	79.13	155.06
20	58.33	55.56	66.67	113.06	65.25
21	47.92	41.67	58.33	217.69	153.50

22	37.50	33.33	33.33	18.56	17.94
23	31.25	52.78	66.67	248.38	72.19
24	62.50	44.44	50.00	176.63	104.25
25	79.17	52.78	41.67	136.94	128.63
26	39.58	55.56	58.33	256.13	22.50
27	27.08	38.89	33.33	283.00	229.56
28	31.25	47.22	25.00	114.31	15.38
29	45.83	58.33	50.00	31.13	31.81
30	60.42	72.22	75.00	-79.44	-1.50
Mean	50.42	53.52	51.94	154.09	101.20
SD	16.30	13.28	17.96	106.99	82.84
SE	3.03	2.47	3.34	19.53	15.12
Lower_95CI	44.23	48.47	45.12	115.80	71.55
Upper_95CI	56.61	58.56	58.77	192.38	130.84

The TFEQ-18 raw scores were standardized to a 0–100 % scale [(raw-score-lowest possible raw score)/ raw score range] x 100. Higher scores indicate greater CR, UE, and EE.

Appetite and cravings-related measures were assessed at multiple time points (t-pre, t0, t30, t60, t120, t180, t240) using a 100-point online VAS. Appetite suppression scores were calculated as the mean of satiety, fullness, PFC, and (100 − hunger). Cravings suppression scores were calculated as the mean of the sweet, salty, savory, and fatty craving items. Both composites were summarized as baseline-adjusted incremental AUC (from t0 to t240, using the trapezoidal method). Higher values reflect a lower appetite/greater satiety, or greater suppression of craving.

Table S4. Genotype distribution of four SNPs related to eating behaviors (N = 30)

ID	Age Oct 20	BMI kg/m²	BMI Status	FTO rs9939609	CD36 rs1761667	MC4R rs17782313	CLOCK rs1801260
1	26	21.6	Normal weight	AA	GA	TT	TC
2	33	22.7	Normal weight	TT	GA	TT	TC
3	29	21.9	Normal weight	TT	GG	TT	TC
4	33	22.3	Normal weight	AA	AA	TT	CC
5	32	24.6	Normal weight	TA	GG	CT	TT

6	27	26.8	Overweight	TT	GA	TT	TT
7	29	22.1	Normal weight	TA	GG	TT	TT
8	26	27.6	Overweight	TA	GA	CT	TT
9	31	24.7	Normal weight	TT	GA	TT	TC
10	28	22.5	Normal weight	TT	GA	CT	TT
11	26	22.6	Normal weight	TT	GG	CT	TT
12	31	24.7	Normal weight	TA	GG	CC	TC
13	22	25.9	Overweight	TT	GG	CT	TT
14	25	24.9	Normal weight	TA	GA	TT	TT
15	26	23.8	Normal weight	AA	GA	CT	TT
16	25	25.2	Overweight	TT	GA	TT	TC
17	30	29.1	Overweight	TA	GA	CT	TT
18	22	22.6	Normal weight	AA	GG	CC	TT
19	23	23.3	Normal weight	TA	GG	CT	TT
20	28	31.3	Obese	TA	AA	TT	TC
21	32	23.3	Normal weight	TT	GA	TT	TT
22	25	25.3	Overweight	TT	AA	TT	CC
23	28	22.3	Normal weight	TT	GA	CT	TT
24	23	29.5	Overweight	TT	GG	TT	TT
25	21	25.6	Overweight	TT	GG	CT	TT
26	25	24.1	Normal weight	TA	GA	TT	TC
27	30	20.3	Normal weight	TT	GG	CT	TT
28	34	23.7	Normal weight	TT	GA	CT	TT
29	30	21.6	Normal weight	TA	GA	CT	TT
30	31	29.2	Overweight	AA	AA	TT	TC
Mean	27.7 ± 3.6	24.5 ± 2.7					
Allele Freq				T= 0.67	G = 0.62	T = 0.72	T = 0.78
Allele Freq				A = 0.33	A = 0.38	C = 0.28	C = 0.22
Total				15 = TT	11 = GG	15 = TT	19 = TT
Total				10 = TA	15 = GA	13 = TC	9 = TC

Total			5 = AA	4 = AA	2 = CC	2 = CC

Genotypic data for four SNPs (FTO rs9939609, CD36 rs1761667, MC4R rs17782313, CLOCK rs1801260) are presented with individual demographic information. BMI categories were defined according to World Health Organization criteria: normal weight (18.5–24.9 kg/m²), overweight (25.0–29.9 kg/m²), and obesity (≥ 30.0 kg/m²). Allele frequencies and genotype counts were calculated for each SNP.

Table S5. Hardy-Weinberg Equilibrium (HWE) and chi-square calculations for four SNPs (N = 30)

Eating behavior-related SNPs	*p* (Allele 1 Freq) from N30	q (Allele 2 Freq) from N30	Expected Ref Homozygote 1 (p^2 * N)	Expected Alt Heterozygote (2pq * N)	Expected Alt Homozygote 2 (q^2 * N)	Chi-Square
FTO rs9939609	0.667	0.333	13.35	13.33	3.33	0.350
CD36 rs1761667	0.617	0.383	11.42	14.18	4.40	0.926
MC4R rs17782313	0.717	0.283	15.42	12.17	2.40	0.930
CLOCK rs1801260	0.783	0.217	18.39	10.19	1.41	0.577

Genotypes were coded as 0, 1, and 2, p-value > 0.05: No significant deviation from HWE, suggesting the population is likely in equilibrium.

Table S6. Example Benjamini–Hochberg (BH) FDR calculation for FTO (5 outcomes, α = 0.05)

Rank (i)	Raw *p* value	BH cutoff $(i/m) \times \alpha$	Significant	Notes
1	0.0067* EE	0.010	Yes	Emotional eating passes
2	0.240 UE	0.020	No	Above cutoff
3	0.284 AS	0.030	No	Above cutoff
4	0.610 CS	0.040	No	Above cutoff
5	0.610 CR	0.050	No	Above cutoff

BH for FTO-5 outcomes (m = 5, α = 0.05): Formula: (i / m) × α, i = the rank, m = number of tests (5), α = chosen FDR level (0.05). Significant for FTO*. remains significant (adjusted 0.0067 x 5 = $p \approx 0.034$).

Table S7. Example Benjamini–Hochberg (BH) FDR calculation for CLOCK (5 outcomes, α = 0.05)

Rank (i)	Raw *p* value	BH cutoff $(i/m) \times \alpha$	Significant	Notes
1	0.013 CS	0.010	No	Above cutoff
2	0.387 UE	0.020	No	Above cutoff
3	0.424 EE	0.030	No	Above cutoff
4	0.452 CR	0.040	No	Above cutoff
5	0.891 AS	0.050	No	Above cutoff

The BH decision rule uses a cutoff $(i/m) \times \alpha$with $m = 5$. None of the CLOCK outcomes pass. The smallest p (0.013 for cravings suppression) yields BH-adjusted q ≈ 0.065, which is > 0.05.

Table S8. Effect size interpretation (%Δ per allele relative to the outcome mean)

SNP	Cognitive restraint (%)	Uncontrolled eating (%)	Emotional eating (%)	Cravings suppression (%)	Appetite suppression (%)
FTO rs9939609	+4.22	+7.36	+22.47	−10.47	−18.51
CD36 rs1761667	−11.94	+0.34	+10.36	−35.48	−13.81
MC4R rs17782313	+10.04	+2.95	+2.87	+29.91	−1.19
CLOCK rs1801260	−7.50	−6.61	−8.51	−58.47	−2.89

%Δ per allele = (β unstd / outcome mean) × 100 (how much the outcome changes for each additional minor allele using the unadjusted model (Cole, 2000). Outcome means used were CR 50.42; UE 53.52; EE 51.94; appetite suppression 154.09; cravings suppression 101.20.
